# Supplementary material for: Dissecting gene regulatory networks governing human cortical cell fate
Source: Nature. 2026 Jan 21;651(8106):732–42. doi: 10.1038/s41586-025-09997-7 (PMC12999477; doi:10.1038/s41586-025-09997-7)
Supplement: Supplementary file 2 — Reporting Summary [file 41586_2025_9997_MOESM2_ESM.pdf]

nature portfolio

Corresponding author(s): Alex A. Pollen

Last updated by author(s): Oct 14, 2025

Reporting Summary

Nature Portfolio wishes to improve the reproducibility of the work that we publish. This form provides structure for consistency and transparency in reporting. For further information on Nature Portfolio policies, see our Editorial Policies and the Editorial Policy Checklist.

Statistics

For all statistical analyses, confirm that the following items are present in the figure legend, table legend, main text, or Methods section.

n/a	Confirmed	
☐	☒	The exact sample size (*n*) for each experimental group/condition, given as a discrete number and unit of measurement
☐	☒	A statement on whether measurements were taken from distinct samples or whether the same sample was measured repeatedly
☐	☒	The statistical test(s) used AND whether they are one- or two-sided<br>*Only common tests should be described solely by name; describe more complex techniques in the Methods section.*
☐	☒	A description of all covariates tested
☐	☒	A description of any assumptions or corrections, such as tests of normality and adjustment for multiple comparisons
☐	☒	A full description of the statistical parameters including central tendency (e.g. means) or other basic estimates (e.g. regression coefficient) AND variation (e.g. standard deviation) or associated estimates of uncertainty (e.g. confidence intervals)
☐	☒	For null hypothesis testing, the test statistic (e.g. *F*, *t*, *r*) with confidence intervals, effect sizes, degrees of freedom and *P* value noted<br>*Give P values as exact values whenever suitable.*
☒	☐	For Bayesian analysis, information on the choice of priors and Markov chain Monte Carlo settings
☒	☐	For hierarchical and complex designs, identification of the appropriate level for tests and full reporting of outcomes
☐	☒	Estimates of effect sizes (e.g. Cohen's *d*, Pearson's *r*), indicating how they were calculated

Our web collection on statistics for biologists contains articles on many of the points above.

Software and code

Policy information about availability of computer code

Data collection	Software used included Thermo Fisher EVOS M7000 Software v2.2.804.158, BD FACSDiva software V9.0, Illumina sequencer control software (NovaSeq) and bcl2fastq (bcl2fastq2) software.
Data analysis	Code used for data analysis is available at https://github.com/jding5066/perturbTF and https://github.com/cnk113/NextClone. Analytical details can be found in the Methods section in the manuscript.<br>Packages used to analyze scRNA-seq data include: Cellranger v7.0.2, DRAGEN Single Cell RNA v4.4.5, seacells v0.3.3, SCENIC+ v1.0.1.dev4 +ge4bdd9f, Vireo v0.5.8, Scanpy v1.9.6, SCVI-tools v0.20.0, Pertpy v0.5.0, pyDEseq2 v0.4.10, Milo v2.0.0, scLiTr v0.1.4, Monocle3 v1.3.7, CoSpar v0.3.3, scFates v1.0.9, pathFindR v2.4.1.9001, scVelo v0.3.1, velocyto v0.17.17, DCATS v1.2.0.<br>Image processing was performed using Fiji/ImageJ v2.14.0 and flow data analysis was performed using Flowjo v10.10.0.

For manuscripts utilizing custom algorithms or software that are central to the research but not yet described in published literature, software must be made available to editors and reviewers. We strongly encourage code deposition in a community repository (e.g. GitHub). See the Nature Portfolio guidelines for submitting code & software for further information.

Data

Policy information about availability of data

All manuscripts must include a data availability statement. This statement should provide the following information, where applicable:

- Accession codes, unique identifiers, or web links for publicly available datasets
- A description of any restrictions on data availability
- For clinical datasets or third party data, please ensure that the statement adheres to our policy

Raw sequencing data for macaque and processed lineage tracing data for both species are deposited on GEO accession number: GSE284197.
Raw sequencing and processed data for human specimens are available through dbGaP under accession number phs002624.v5.p1.

Research involving human participants, their data, or biological material

Policy information about studies with human participants or human data. See also policy information about sex, gender (identity/presentation), and sexual orientation and race, ethnicity and racism.

Reporting on sex and gender	Sex and Gender were not used as selection criteria for sample collection. Sex of de-identified samples were determined based on PCR-based on genotyping and sex-specific gene expression. Male and female samples were treated equally.
Reporting on race, ethnicity, or other socially relevant groupings	No race, ethnicity, or other socially relevant groupings were performed in this study.
Population characteristics	De-identified human brain samples from gestational week 16 to 23 were used for this study and listed in Supplementary Table 3 and 6. No population characteristics other than age were used in the data analysis.
Recruitment	No recruitment criteria were used. De-identified tissue samples were collected from previous patient consent in strict observance of the legal and institutional ethical regulations, which was performed by the clinic.
Ethics oversight	Human Gamete, Embryo, and Stem Cell Research Committee (institutional review board) at the University of California, San Francisco.

Note that full information on the approval of the study protocol must also be provided in the manuscript.

Field-specific reporting

Please select the one below that is the best fit for your research. If you are not sure, read the appropriate sections before making your selection.

☒ Life sciences ☐ Behavioural & social sciences ☐ Ecological, evolutionary & environmental sciences

For a reference copy of the document with all sections, see nature.com/documents/nr-reporting-summary-flat.pdf

Life sciences study design

All studies must disclose on these points even when the disclosure is negative.

Sample size	4 individuals were collected for the initial Perturb-seq. Validation experiments focusing on top candidates were carried out using additional 13 individuals (2D single cell lineage tracing) in both human and macaque, 4 human individuals in organotypic slice culture and 9 individuals (flow cytometry) in human. Data were collected from as many individuals as were available. Downsampling was performed and showed in extended data fig. 3 and 4 to show sufficiency of sample sizes.
Data exclusions	Cells assigned to sgRNAs with knockdown efficiency lower than 75% was excluded from this study to ensure recovery of knockdown phenotypes. For lineage tracing analysis, clones lower than 3 cells and clones with conflicted sgRNA assignments were excluded from downstream analysis to consider multicellular clones labeled at the progenitor stage and expanded during differentiation to study effects of TF perturbations on progenitor lineage.
Replication	In order to improve replicability, samples for initial Perturb-seq were collected from 4 different individuals spanning from gestational week (GW)16-18. For lineage resolved targeted screen, 13 human individuals(GW17-22) and 4 macaque (PCD60-80) individuals and organotypic slice culture were used to ensure reproducibility of findings from the initial screen. Furthermore, flow cytometry was used to corroborate findings from different differentiation timepoints across 9 human individuals. Phenotypes of ARX, NR2E1 and ZNF219 KD reported in the study were consistently observed across batches, species and modalities.
Randomization	Randomization was not relevant to this study. Samples were pooled and treated equally between perturbation conditions.
Blinding	Blinding was not relevant to this study. Data derived from pooled screens were analyzed based on on unbiased computational pipeline.

Reporting for specific materials, systems and methods

We require information from authors about some types of materials, experimental systems and methods used in many studies. Here, indicate whether each material, system or method listed is relevant to your study. If you are not sure if a list item applies to your research, read the appropriate section before selecting a response.

Materials & experimental systems

n/a	Involved in the study
☐	☒ Antibodies
☐	☒ Eukaryotic cell lines
☒	☐ Palaeontology and archaeology
☒	☐ Animals and other organisms
☒	☐ Clinical data
☒	☐ Dual use research of concern
☒	☐ Plants

Methods

n/a	Involved in the study
☒	☐ ChIP-seq
☐	☒ Flow cytometry
☒	☐ MRI-based neuroimaging

Antibodies

Antibodies used

mouse-EOMES (Thermofisher, 14-4877-82, 2288573)
rabbit-NEUROD2 (Abcam, ab104430, GR3414328-2)
goat-SOX9 (R&D, AF3075, WIL0421041)
mouse-DLX2 (Santa Cruz, sc-393879, C0424)
mouse-HOPX (Santa Cruz, sc-398703, C0823)
mouse-KI67 (BD, 550609, 2052205)
sheep-ARX (R&D, AF7068SP, CFOM0225031)
rabbit-SCGN (Millipore-sigma, HPA006641)
rabbit-KI67 (Vector, VP-K451)
KI67-421 (BD, 565929, 4282091)
SOX2-PerCP-Cy5.5 (BD, 561506, 3313075)
EOMES-PE-Cy7 (Invitrogen, 25-4877-42, 2510765)
donkey anti-mouse-488(Thermo, A32766, WF319853)
donkey anti-rabbit-647 (Thermo, A32795, WA308388)
donkey anti-sheep-647 (Thermo, A21448, 2045339)
donkey anti rabbit 555 (Thermo, A32794, WG322207)

Validation

All primary antibodies used in this study have been validated by the manufacturer to be suitable for the respective application (ICC, IHC and Flow) against human protein. Antibodies used for ICC and IHC were tested in fixed human tissue and showed expected anatomical distribution and subcellular localization. No off-target staining was observed.
All secondary antibodies were pre-adsorbed to minimize cross-reactivity and validated by the manufacturer. No reaction was observed against serum proteins of other species.

Eukaryotic cell lines

Policy information about cell lines and Sex and Gender in Research

Cell line source(s)

Lenti-X HEK293T (Takara Bio, 632180) were used for lentiviral production.
Primary cell culture from a total of 18 humans (8 females, 5 males, 5 unknown) and 4 macaques (2 female and 2 males) were derived from cryopreserved tissue chunks from the developing cortex were used in this study. Organogtypic slice culture were derived from 4 humans (1 female and 3 males). De-identified human tissue samples were collected with previous patient consent in strict observance of the legal and institutional ethical regulations. Protocols were approved by the Human Gamete, Embryo, and Stem Cell Research Committee (institutional review board) at the University of California, San Francisco. The Primate Center at the University of California, Davis, provided 4 specimens of cortical tissue from PCD60 (n=1), PCD75 (n=1) and PCD80 (n=2) macaques. All animal procedures conformed to the requirements of the Animal Welfare Act, and protocols were approved before implementation by the Institutional Animal Care and Use Committee at the University of California, Davis.

Authentication

HEK293T cells were authenticated by the vendor (Takara Bio) and morphology.

Mycoplasma contamination

HEK293T and primary cell culture was tested and found negative for mycoplasma infection.

Commonly misidentified lines
(See ICLAC register)

No commonly misidentified cell lines were used in the study.

Plants

Seed stocks	*Report on the source of all seed stocks or other plant material used. If applicable, state the seed stock centre and catalogue number. If plant specimens were collected from the field, describe the collection location, date and sampling procedures.*
Novel plant genotypes	*Describe the methods by which all novel plant genotypes were produced. This includes those generated by transgenic approaches, gene editing, chemical/radiation-based mutagenesis and hybridization. For transgenic lines, describe the transformation method, the number of independent lines analyzed and the generation upon which experiments were performed. For gene-edited lines, describe the editor used, the endogenous sequence targeted for editing, the targeting guide RNA sequence (if applicable) and how the editor was applied.*
Authentication	*Describe any authentication procedures for each seed stock used or novel genotype generated. Describe any experiments used to assess the effect of a mutation and, where applicable, how potential secondary effects (e.g. second site T-DNA insertions, mosiacism, off-target gene editing) were examined.*

Flow Cytometry

Plots

Confirm that:

☒ The axis labels state the marker and fluorochrome used (e.g. CD4-FITC).

☒ The axis scales are clearly visible. Include numbers along axes only for bottom left plot of group (a 'group' is an analysis of identical markers).

☐ All plots are contour plots with outliers or pseudocolor plots.

☒ A numerical value for number of cells or percentage (with statistics) is provided.

Methodology

Sample preparation	Cryopreserved tissue chunks of developing human cortex were dissociated with papain (Worthington Biochemical Corporation, LK003153) and plated for culture at a density of 500,000 cells/cm2. Cells were expanded for 12 days, transduced with all-in-one CRISPRi vector on day 5 and differentiated for 7 days.Cell culture was dissociated with Accutase (STEMCELL Technologies, 07920) supplemented with 5% Trehalose (Fisher Scientific, BP268710) and fixed in Foxp3 fixation buffer for 30 min at room temperature. Cells were then washed twice with Foxp3 permeabilization buffer and then stained with primary antibodies mouse-DLX2 (Santa Cruz Biotechnology, sc-393879) and rabbit-NEUROD2 (Abcam, ab104430) at 1:100 dilution. After washing with Foxp3 permeabilization buffer, cells were stained with secondary antibodies donkey anti-mouse-488 and donkey anti-rabbit-647 (Invitrogen) at 1:200. Finally, cells were stained with conjugated antibodies KI67-421 (BD, 562899), SOX2-PerCP-Cy5.5 (BD, 561506), EOMES-PE-Cy7 (Invitrogen, 25-4877-42) at 1:100, washed twice with Foxp3 permeabilization buffer and resuspended in 0.2% BSA in PBS.
Instrument	BD LSRFortessa
Software	BD FACSDiva was used for data collection and Flowjo was used for analysis.
Cell population abundance	Cells were stained for nuclear markers KI67-421, SOX2-PerCP-Cy5.5, DLX2-488, NEUROD2-647 and EOMES-PE-Cy7. Populations include heterogeneous progenies derived from cortical radial glia which varies between individual, stage, differentiation timepoints and knockdown conditions.
Gating strategy	FSC/SSC values were used to filter debris and doublets, resulted singlets were used for downstream gating. Positive and negative populations for each marker were identified based on the bimodal distribution of marker expression.

☒ Tick this box to confirm that a figure exemplifying the gating strategy is provided in the Supplementary Information.
